# Risk of ptosis following eyelid speculum assisted intravitreal anti-VEGF injections

**DOI:** 10.1007/s00417-025-06763-3

**Published:** 2025-02-13

**Authors:** Efraim Berco, Michael Ostrovsky, Obinna Esomchukwu, Ortal Zaks, Mor Schlesinger, Elkin Jose Cervantes Molina, Shalhevet Goldfeather Ben-Zaken, Nir Shoham-Hazon

**Affiliations:** 1 Department of Ophthalmology, Kaplan Medical Center, Faculty of Medicine, The Hebrew University of Jerusalem, Jerusalem, Israel; 2 Dalhousie University Medical School, Halifax, NS Canada; 3 Miramichi Eye NB Centre of Excellence, Miramichi, NB Canada

**Keywords:** Anti-VEGF, Blepharoptosis, Eyelid speculum, Intravitreal injection, Margin-reflex distance

## Abstract

**Purpose:**

Intravitreal injections are essential for treating retinal diseases. This study aims to assess the impact of repeated intravitreal anti-VEGF injections using an eyelid speculum on the risk of ptosis development.

**Methods:**

This single-center, retrospective chart review included 114 patients (228 eyes) who received at least three unilateral intravitreal anti-VEGF injections. Patient demographics, clinical characteristics, and MRD1 and MRD2 of the injected and the fellow eyes were analyzed. A multivariate linear regression model was constructed to identify predictors of MRD1 in the injected eye.

**Results:**

The study cohort had a mean age of 75.18 ± 0.98 years, with 57% female patients. On average, patients received 16.92 ± 1.18 injections. At the final follow-up, no significant difference was observed in mean MRD1 between injected and fellow eyes (2.85 ± 0.11 mm vs. 2.90 ± 0.11 mm, *p* = 0.445). Multivariate regression analysis identified MRD1 of the fellow eye as the only significant predictor of MRD1 in the injected eye (β = 0.769, *p* < 0.001).

**Conclusion:**

The repeated use of an eyelid speculum during intravitreal anti-VEGF injections does not significantly contribute to ptosis development. MRD1 tends to be similar between the injected and non-injected eye, suggesting that intrinsic factors may play a more crucial role in determining eyelid position than the mechanical effects of the procedure.

**Supplementary Information:**

The online version contains supplementary material available at 10.1007/s00417-025-06763-3.

## Abstract (highlights)

**Key messages:**

*****What is known***:**

- Ophthalmic surgery can contribute to blepharoptosis development, likely due to compressive mechanical trauma to various eyelid structures due to eyelid speculum use.
- Intravitreal anti-VEGF injections are common, often repetitive procedures which also involves the use of an eyelid speculum, yet data regarding their association with blepharoptosis is scarce.

*****What is new***:**

- This study found no significant differences in eyelid position between patients’ injected and non-injected eyes following repeated anti-VEGF injections.
- Our analysis showed that intrinsic eyelid factors, rather than injection-related, likely play a more significant role in eyelid position post-injection.
- Findings suggest that routine eyelid speculum use during injections does not increase the risk of blepharoptosis.

**Supplementary Information:**

The online version contains supplementary material available at 10.1007/s00417-025-06763-3.

## Introduction

Intravitreal injections (IVI) have become a cornerstone in the management of various retinal diseases, offering significant improvements in prognosis for patients with conditions such as age-related macular degeneration (AMD), diabetic macular edema (DME), and retinal vein occlusion (RVO). They are, to date, the most frequently performed intraocular procedure globally [1]. Furthermore, the advancement of early detection technologies, the expanding list of intravitreal agents and medical indications, and the aging population are driving forces behind a further increase in the annual use of IVI [2, 3]. Despite their proven efficacy and safety profile, including a very low risk of severe complications such as endophthalmitis or retinal detachment, concerns regarding potential side effects persist [3, 4].

One such concern involves mechanical trauma to the eyelids due to the use of an eyelid speculum prior to injection, potentially resulting in blepharoptosis. Blepharoptosis, or drooping of the upper eyelid, is characterized by a decrease in margin-reflex distance (MRD1). Clinically, this condition may vary from a cosmetic nuisance to severe visual dysfunction with a constriction of the superior visual field [5, 6]. Mechanical ptosis has previously been described following various ophthalmological procedures, including cataract, glaucoma, vitreoretinal, and refractive ocular surgery [7–13]. One prospective study [14], evaluating eyes receiving panretinal photocoagulation, found ptosis in nearly 10% of treated eyes. In these reports, ptosis is thought to result at least in part from dehiscence of the levator palpebrae superioris aponeurosis, which may be associated with the use of an eyelid speculum [15, 16]. It has also been postulated that sustained compressive forces from the speculum can damage upper eyelid myo-neural or myo-vascular connections, leading to ptosis due to the resultant muscle injury [17].

In IVI, an eyelid speculum is used to prevent contact between the injection needle and the eyelashes or the lid margins. Often, speculum application is described by patients as one of the more unpleasant aspects of IVI [18]. Although IVI typically involve only brief exposure to the eyelid, the repetitive nature of this procedure over months and years raises concerns regarding cumulative eyelid trauma and its impact on the risk of ptosis development.

To date, there has been a paucity of data published regarding mechanical ptosis in patients treated with IVI, particularly with anti-VEGF agents (Supplemental Table 1). Therefore, this study aims to investigate whether repeated IVI with an eyelid speculum are associated with measurable changes in MRD1 and to identify factors that might predict such changes.

## Methods

This single-center, retrospective chart review was conducted at the Miramichi Eye Centre of Excellence in Miramichi, New Brunswick, Canada. The study and all procedures involving human participants were in accordance with the ethical standards of the Horizon Health Network Research Ethics Board (File No. 101865) and with the 1964 Helsinki declaration and its later amendments or comparable ethical standards.

### Study population

The study included all consecutive patients who received a minimum of three unilateral intravitreal anti-VEGF injections for AMD or DME between January 2011 and December 2023, with a minimum follow-up of at least 12 months.

The following exclusion criteria were applied:

1. Patients with pre-existing blepharoptosis or a history of eyelid surgery.
2. Patients who received bilateral intravitreal injection therapy.
3. Patients who underwent ocular surgery other than cataract extraction, or whose cataract surgery was performed within 12 months before their first intravitreal anti-VEGF injection.

### Intravitreal injection technique

Topical anaesthesia drops (Oxybuprocaine hydrochloride 0.4%) were administered to the eye. A disposable sterile stainless steel all-wire “Barraquer” eyelid speculum was then inserted. The periocular area, including the eyelids and lashes, was thoroughly cleaned using a 5% povidone-iodine solution. Several drops of the solution were instilled directly into the conjunctival sac to disinfect the ocular surface. In cases where patients had an iodine allergy, fourth-generation fluoroquinolone drops were applied for 4 days before and after the injection. After a one-minute waiting period, a 32-gauge needle was used to slowly inject 0.05 ml of the anti-VEGF drug solution into the vitreous cavity, 3.5 to 4.0 mm posterior to the limbus, depending on lens status. The needle was then withdrawn and a cotton swab was applied to the injection site to prevent reflux of the medication. This protocol was consistently applied across all cases to ensure uniformity in the procedure.

### Assessment of Margin-Reflex Distance (MRD)

MRD was defined as the distance between the corneal light reflex and the upper (MRD1) or lower (MRD2) lid margin, measured in millimetres (mm). The operational definition of ptosis was an MRD1 of 2.00 mm or less. One investigator (NSH) acquired standardized photographs of the eyes of patients fitting the inclusion criteria, with informed patient consent, during a routine clinic follow-up visitation. For each patient, this was defined as the final follow-up timepoint. MRD1 and MRD2 measurements were independently assessed by two trained investigators (OZ and MS). The investigators were blinded to all relevant clinical information, including the frequency and laterality of IVI. A third investigator (OE) collected and organized the MRD data, which was then compared between the injected and non-injected (fellow) eyes.

### Data collection

The electronic medical records of eligible patients were reviewed to extract data on patient demographics and clinical characteristics, including age, sex, previous ocular surgery, lens status (phakic/pseudophakic/aphakic), as well as the number, laterality, and final interval between injections received.

### Statistical analysis

Statistical analysis was performed using SPSS software (Version 27). A *p*-value of < 0.050 was considered statistically significant for all tests. Descriptive statistics, including means and standard error of the mean for continuous variables and frequencies for categorical variables, were calculated. Data distribution was assessed using Shapiro–Wilk tests of normality. Comparative outcomes of continuous variables between the injected and fellow eyes were analyzed using paired t-tests for normally distributed variables and Wilcoxon Signed-Rank tests for non-normally distributed variables. A multivariate linear regression model was constructed to predict MRD1 of the injected eye, including the following confounding factors: age, gender, eye laterality, lens status, number of injections, the interval between the two final injections, MRD2 of the injected eye, MRD1 and MRD2 of the fellow eye.

## Results

The study included 114 patients (228 eyes) who underwent unilateral intravitreal anti-VEGF injections. Sixty-five patients (57%) were female, with a mean age of 75.18 ± 0.976. The injected eye was the right eye in 58 (50.9%) of patients. On average, patients received 16.92 ± 1.18 injections (median 10, range 1–75). The final injection interval was 8.92 ± 0.48 weeks. The lens status of the injected eye was phakic in 55 patients (48.2%). Table 1 provides a summary of the cohort’s baseline demographic and clinical characteristics.

**Table 1:** Baseline demographics and clinical characteristics

Variables	Patients (*n* = 114)
Age (years)	75.18 ± 0.98
Sex (female)	65 (57.0%)
Injected eye (right)	58 (50.9%)
Injections (n)	16.92 ± 1.18
Final injection interval (weeks)	8.92 ± 0.48
Injected eye lens status (phakic)	55 (48.2%)

Values are presented as counts (percentages) or means ± standard error of the mean

**Fig. 1:**
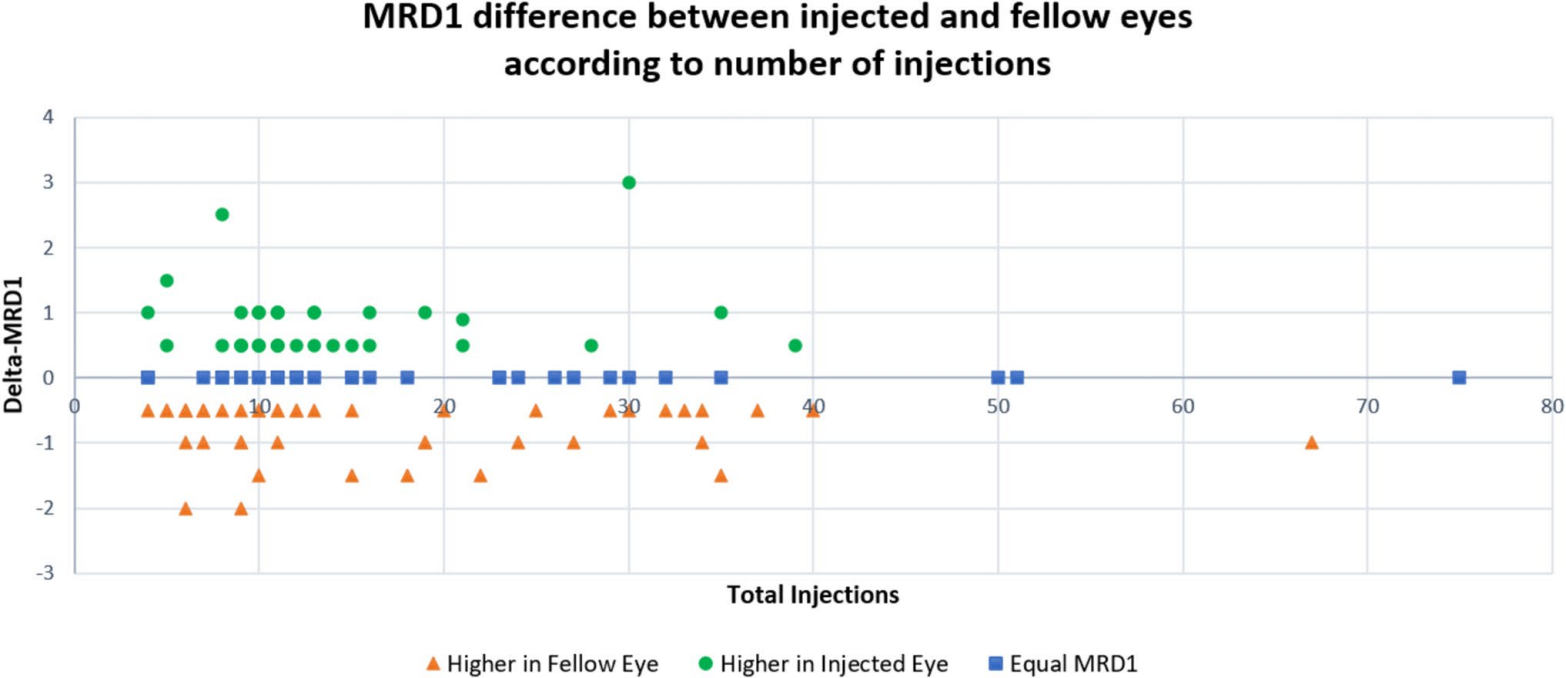
**MRD1 difference between injected and fellow eyes according to number of injections.** The difference in MRD1 (mm) between the injected eye and the fellow eye of the same patient, according to the total number of injections received. Patients are categorized into three groups: orange triangles indicate cases where the fellow eye had a higher MRD1, green circles indicate that the injected eye had higher MRD1, and blue squares indicate equal MRD1 between the eyes. Each group comprises approximately one-third of the patients

When comparing the injected eyes with the fellow eyes at the final follow-up, ptosis (defined as having an MRD1 of 2.00 mm or less) was observed in 35 (30.7%) injected eyes and in 33 (28.9%) fellow eyes (*p* = 0.772). There were no significant differences in mean MRD1 (2.85 ± 0.11 vs. 2.90 ± 0.11 mm, *p* = 0.445) or mean MRD2 (5.02 ± 0.11 vs. 5.00 ± 0.11 mm, *p* = 0.993). To further evaluate potential differences, the delta between MRD measurements of the injected and the fellow eyes was calculated for each patient. The mean delta-MRD1 was −0.05 ± 0.08, and the mean delta-MRD2 was 0.02 ± 0.06. Overall, MRD1 was lower in the injected eye in 45 (39.5%) patients, equal in both eyes 33 (28.9%) patients, and lower in the fellow eye in 36 (31.6%) patients. Figure 1 demonstrates the delta-MRD1 according to total number of injections.

Further analysis using a multivariate linear regression model was conducted to predict MRD1 in the injected eye. The model included the following factors: age, gender, eye laterality, lens status, number of injections, interval (weeks) between the final two injections, injected eye MRD2 and fellow eye MRD1 and MRD2. The only significant predictor of MRD1 in the injected eye was the MRD1 of the fellow eye, with a strong positive association (unstandardized β coefficient of 0.769, *p* < 0.001). Table 2 summarizes the results of the regression analysis.

**Table 2:** Linear regression analysis predicting MRD1 in the injected eye

Variable	β coefficient	*p*-value
Age (years)	−0.006	0.425
Gender (female)	0.007	0.963
Eye laterality (right)	0.056	0.710
Lens status (phakic)	−0.110	0.508
Injections (n)	−0.008	0.210
Last injection interval (weeks)	0.006	0.593
Injected eye MRD2	0.014	0.906
Fellow eye MRD1	**0.769**	** < 0.001**
Fellow eye MRD2	0.017	0.885

Presented are the unstandardized β coefficient and p-values for the multivariate linear regression model. The analysis evaluates factors predicting the MRD1 of patients’ injected eye. The model is adjusted for all baseline demographic and clinical characteristics, as well as the MRD1 of the fellow eye and MRD2 of the injected and fellow eyes

*MRD* Margin-reflex distance

## Discussion

Eyelid speculum use during various ophthalmological procedures is a well-known risk factor for post-operative ptosis, likely by weakening the levator palpebrae superioris muscle [15, 19]. This phenomenon is attributed to the prolonged mechanical stress exerted on the eyelid during surgery. However, it has not been previously investigated whether the brief, intermittent use of an eyelid speculum during anti-VEGF IVI, repeated over months or years, could similarly cause ptosis. It is an especially pertinent concern, as anti-VEGF IVI are already among the most commonly performed medical procedures worldwide, with their frequency steadily increasing [2, 3]. To our knowledge, this is the first study to assess the potential risk of injection-related ptosis by comparing MRD1 measurements in patients who underwent unilateral anti-VEGF IVI over 12 months with those of their non-injected fellow eyes.

Our findings demonstrated no significant association between repeated IVI using an eyelid speculum and the development of blepharoptosis as compared to the patients’ fellow eye. Additionally, no dose–response effect was observed, as there was no trend in MRD1 difference based on the number of injections received. This finding was further corroborated by the multivariate linear regression analysis, where the number of injections was not a significant predictor of MRD1 (unstandardized β coefficient = −0.008, *p* = 0.210). Rather, MRD1 of the fellow eye was the only significant predictor of the MRD1 in the injected eye, suggesting that eyelid position is more likely related to patients’ intrinsic factors than to the external mechanical forces exerted during the IVI procedure.

Similar findings were recently reported by Lee et al. [20], who showed that neither anti-VEGF IVI, nor the use of an eyelid speculum during injections in particular, carried significantly increased ptosis risk. However, it is important to note that their study relied on ICD-10 diagnostic codes to identify cases of ptosis following the visit in which an AMD diagnosis code was first recorded in their chart. This method risks significant information bias, as most clinicians do not routinely assess MRD1 in the absence of significant, clinically apparent, and/or symptomatic ptosis. As such, the use of diagnostic coding for patient selection may result in an underestimation of mild ptosis. Our study, in contrast, directly assessed blepharoptosis by measuring the MRD of both injected and fellow eyes in a cohort of consecutive, injection-naive patients treated with anti-VEGF IVI. Hence, it more thoroughly investigated fine changes in eyelid position, as opposed to cases of gross ptosis. Additionally, 11 of the 18 patients who developed ptosis in the injection group in their study developed ptosis bilaterally, despite only being injected unilaterally, 4 of the 18 patients had not received IVI for 12 months or longer prior to the onset of ptosis, and 2 of the 18 patients had received intravitreal triamcinolone injections in the 6 months prior to ptosis onset, which were previously associated with a risk of ptosis development due to direct myopathic effects of the solvent agent used in these injections [21–23]. All of these factors suggest that ptosis in their study may have been caused by aetiologies unrelated to IVI using an eyelid speculum.

### Eyelid speculum use

Eyelid speculum use during ocular procedures has long been implicated as a contributing factor to ptosis development, due to the mechanical stress it places on eyelid muscles [24]. As IVI are comparatively brief procedures, the main concern is that the cumulative effect of repeated eyelid speculum use could similarly lead to ptosis. However, the results of our study challenge this assumption. Although we observed a relatively high prevalence of ptosis (approximately one-third of our patients), this was consistent in both injected and non-injected eyes, indicating that speculum use was unlikely to be the cause.

During ocular surgery, the eyelid is held open for a significantly longer period than during IVI, potentially increasing the risk of levator muscle dysfunction. To date, there is no consensus in the literature regarding the association between operative time and the risk of post-operative ptosis development. It should be noted, however, that while the existing literature examined this association, it was only studied between differing ocular surgery types, while short exposures to an eyelid speculum (such as with IVI), were not previously examined [7, 11, 25].

The amount of force exerted on patients’ eyelids during the injection may also play a crucial role regarding the risk of ptosis. A previous study [15] found that different speculum types exert varying amounts of force on patients’ eyelids, with reusable speculums exhibiting significantly greater stiffness as compared to disposable speculums. Additionally, their study showed that a narrow palpebral aperture also results in greater forces applied by the speculum, due to the need for greater eyelid displacement. In a study by Linberg et al. [8] on ptosis following radial keratotomy, the authors theorized that the use of rigid speculae, as opposed to flexible “all-wire” speculae, may be partly at fault. They further suggest that, if a rigid speculum is required for the procedure, lid akinesia using anaesthesia may limit aponeurotic damage. This suggestion seems to be supported by reports of lower post-operative ptosis incidence following ocular surgery performed under general anaesthesia, compared with local anaesthesia [26, 27]. One possible reason for this might be the use of muscle relaxants in general anaesthesia preventing patients from squeezing their eyelids against a rigid speculum.

When considered collectively, these findings may help explain why ptosis is linked to ocular surgery, but not to anti-VEGF IVI. It should be noted that in many medical centers, smaller, more flexible, disposable eyelid specula are used during IVI, designed only to expose the injection site, prevent blinking, and ensure sterility. Indeed, in our study, a single type of disposable, “all-wire” speculae was used for all patients, which likely contributed to the absence of increased ptosis risk observed in our cohort. In contrast, the speculae used in ocular surgery are typically larger and force the eyelids open more widely to accommodate surgical instruments, resulting in greater eyelid displacement [8, 12, 28].

Similarly, the recent report by Lee et al. [20] found no difference in ptosis rates between non-injected eyes, eyes injected with a speculum, and eyes injected using manual lid retraction (2.3%, 2.0%, and 1.8%, respectively). While subjective, manual lid retraction was previously shown to be associated with less patient discomfort following IVI, perhaps suggesting lower mechanical forces exerted on the eyelids [29]. Thus, although the authors did not indicate the type of speculum used, a similar ptosis rate between manual retraction and speculum-assisted IVI further supports the hypothesis that other, unrelated aetiologies are at play.

### Drug-related effects

While previous studies have shown varying degrees of association between (non-anti-VEGF) IVI and the development of ptosis, our study adds to the growing body of evidence suggesting that IVI do not inherently pose a significant risk in this context. In a study by Minnella et al. [22] ptosis was observed in a subset of patients following triamcinolone acetonide injections, but the effect was minimal and resolved spontaneously. Another publication [21] also described two case reports, in which patients developed 2 mm of upper eyelid ptosis 1- and 2 months after intravitreal triamcinolone acetonide injections. In these cases, however, ptosis progressively worsened over the following 9 months. In contrast, a different study by Minnella et al. [30] saw no increase in post-injection ptosis following intravitreal ganciclovir injection. The discrepancy between these reports suggests that ptosis may be related to the drug-specific steroid effects on eyelid musculature and innervation, rather than to the IVI procedure itself. A study [23], which examined 29 refractory DME patients that received combined high-dose sub-tenon triamcinolone, laser photocoagulation, and anti-VEGF injections, found a postinjection ptosis rate of 17.2%, with a mean time of onset of 6 months (range 3–9 months). The authors suggested the anterior diffusion of the solute or a myopathic effect by the corticosteroids used as the aetiology for ptosis, rather than a mechanical effect due to manipulation of the eyelids. Indeed, sub-tenon triamcinolone injections were previously described to cause orbital fat prolapse with resultant ptosis [31]. In our study, which focused solely on anti-VEGF agents, we did not observe any significant discrepancy in MRD1 between the injected and the fellow eyes, even after a year of repeated injections using an eyelid speculum. This further supports the assumption that any mechanical ptotic effect, if present, is likely negligible.

### Limitations and future research

The primary limitation of our study is its retrospective nature, which inherently limits the ability to control for certain variables. Most notably, we did not have baseline MRD1 measurements for either eye before the initiation of IVI. Hence, while no significant differences were observed in MRD1 between injected eyes and their non-injected fellow eyes after a year of treatment, we cannot definitively rule out the possibility that the injected eyes had higher baseline MRD1 values, which potentially could have masked mild ptosis induced by the speculum. Nevertheless, this limitation is somewhat mitigated by the relatively large sample size of our study, which increases the likelihood that pre-existing differences in eyelid position were evenly distributed between injected and non-injected eyes. Future long-term prospective studies in injection naïve patients are warranted, to further explore the effect of IVI on eyelid function and position. Additionally, while MRD1 is a widely used and reliable measure of eyelid position, it may not capture all aspects of eyelid function and ptosis. Future research could incorporate other measures, such as levator function or eyelid contour analysis, to provide a more comprehensive assessment. Lastly, patients in our study received a varying total number of injections. While we addressed this limitation by conducting an assessment of dose-dependent effects with similar results, it may somewhat underpower our study in regards to frequently injected eyes.

## Conclusions

Our results lead us to conclude that the repeated use of an eyelid speculum during anti-VEGF IVI over 12 months does not significantly contribute to ptosis development. This may be due to the shorter duration and smaller size of the speculum used during IVI, which are likely insufficient to cause muscle injury. The MRD1 of the fellow eye was the only significant predictor of the MRD1 of the injected eye, suggesting that intrinsic factors related to eyelid anatomy and function are more influential in determining eyelid position than the injection procedure itself. These findings provide reassurance regarding the safety of repeated IVI concerning eyelid position. Future prospective studies are warranted to explore the long-term eyelid changes following anti-VEGF injections.

## Supplementary Information

Below is the link to the electronic supplementary material.

Supplementary file1 (XLSX 12 KB)
